# Comparison of DVB-T Passive Radar Simulated and Measured Bistatic RCS Values for a Pilatus PC-12 Aircraft

**DOI:** 10.3390/s22072766

**Published:** 2022-04-03

**Authors:** Peter J. Speirs, Martin Ummenhofer, Christof Schüpbach, Matthias Renker, Peter Wellig, Diego Cristallini, Daniel W. O’Hagan, Michael Kohler, Axel Murk

**Affiliations:** 1 Institute of Applied Physics, University of Bern, 3012 Bern, Switzerland; axel.murk@unibe.ch; 2 Fraunhofer FHR, Fraunhofer Institute for High Frequency Physics and Radar Techniques, Fraunhoferstr. 20, 53343 Wachtberg, Germany; martin.ummenhofer@fhr.fraunhofer.de (M.U.); diego.cristallini@fhr.fraunhofer.de (D.C.); daniel.ohagan@fhr.fraunhofer.de (D.W.O.); michael.kohler@hensoldt.net (M.K.); 3 armasuisse W+T, 3603 Thun, Switzerland; christof.schuepbach@ar.admin.ch (C.S.); matthias.renker@ar.admin.ch (M.R.); peter.wellig@ar.admin.ch (P.W.)

**Keywords:** passive radar, bistatic RCS, RCS simulation, DVB-T, ultrahigh frequency, turboprop aircraft, field measurements, signal processing, CFAR, range-Doppler analysis

## Abstract

Passive radar is a technology that has huge potential for airspace monitoring, taking advantage of existing transmissions. However, to predict whether particular targets can be measured in a particular scenario, it is necessary to be able to model the received signal. In this paper, we present the results of a campaign in which a Pilatus PC-12 single-engine aircraft was measured with a passive radar system relying on DVB-T transmission from a single transmitter. We then present our work to simulate the bistatic RCS of the aircraft along its flight track, using both the method of moments and the shooting and bouncing ray solvers, assess the uncertainty in the simulations, and compare against the measurements. We find that our simulated RCS values are useful in predicting whether or not detection occurs. However, we see poor agreement between simulated and measured RCS values where measurements are available, which we attribute primarily to the difficulties in extracting RCS measurements from the data and to unmodeled transmission and received path effects.

## 1. Introduction

The research and development in the field of passive radar has been significant over the last two decades. Passive radars have now become an established technology, approaching the stages of commercialization [1,2] and certification [3]. As a consequence, the radar community is now investigating advanced applications that can benefit from the particular characteristics of a passive radar system. Among these, the antistealth capability of passive bistatic radar is one of the most appealing. This relies on both the bistatic acquisition geometry and on the low operating frequencies with respect to conventional monostatic active radars. Although such a characteristic is known and reasonable, few works have addressed the problem of experimentally verifying it [4,5,6]. Specifically, these works mostly consider prediction detection capability of a given passive radar system and compare it with experimental detection performance.

Passive radar antistealth capabilities can also be analyzed in terms of bistatic radar cross section (RCS) [7]. That is, we analyze the bistatic RCS signature measured in time for a given target over a specific target trajectory and target attitude. This approach has been proposed as an aid to target tracking as well as a way to address automatic target recognition (ATR) [8,9,10,11,12].

The problem of comparison between simulated and measured RCS for the active monostatic case was addressed in [13,14]; in [15] a comparison between the simulated and measured RCS of specific targets was performed, obtaining a good match. In the pioneer work [16], the issue of calculating the bistatic RCS from monostatic measurements was addressed, along with its limitations. In [17], the authors focused on the main sources of computation and measurement uncertainties, while [18] focused on the mutual information content between monostatic and bistatic measurements. Weinmann [19] addressed the problem of simulating the RCS of a specific target over a wide bandwidth, concluding that this is a difficult task.

In this paper, we focus on the validation of passive-radar-based, bistatic RCS measurements for a specific target. In particular, in June 2015, armasuisse W+T and Fraunhofer FHR conducted a joint measurement campaign exploiting a DVB-T-based passive radar system and a cooperative target, namely a Pilatus PC-12 aircaft. The target response obtained after conventional passive radar processing [20,21] was extracted for several tens of seconds along the flown trajectory. Thanks to an inertial measurement unit (IMU) mounted onboard the target, both target trajectory and target attitude were recorded. The aforementioned target response was then converted into an estimate of the target bistatic RCS by inverting the bistatic radar equation. In doing so, special attention was devoted to compensating for potential sources of error in the derivation of the measured target RCS. Among these, the control of the measurement noise floor at a constant level over time was the most challenging. To validate the bistatic RCS measurements, corresponding sets of simulated bistatic RCS values were calculated for the same trajectory and attitude by using both methods of moments (MoM) and enhanced shooting and boucing ray (SBR+) solvers, applied to a 3D CAD model of the Pilatus PC-12. On the simulation side, an error analysis was conducted to account for RCS variations over coherent integration time and over signal frequency bandwidth, as well as the potential errors introduced by uncertainties in the IMU measurements and possible assumptions about propagation through the atmosphere.

The key goal of this work is to develop an understanding of the capabilities and limitations of electromagnetic bistatic RCS simulations when applied to a real passive radar measurement, and in so doing, gain some insight into the necessary steps. The intention is that such insights can later be applied to simulations of other aircraft, including in cases where a co-operative target is unavailable for measurement and therefore for which simulation is particularly valuable. This could also later be applied to stealth aircraft.

The remainder of this manuscript is organized as follows. In Section 2, we introduce the measurement campaign jointly conducted by armasuisse W+T and Fraunhofer FHR in Switzerland, also describing the experimental passive radar system and the particular scenario configuration. In Section 3, we describe the passive radar signal processing performed to retrieve the measured bistatic RCS values. In Section 4, the simulation method used to obtain the predicted target bistatic RCS is described. The uncertainties in the simulation method are analyzed in Section 5. The measurement and simulation results are compared in Section 6. Finally, in Section 7, we draw our conclusions.

## 2. Measurement Campaign

In this section, the field trials jointly conducted by armasuisse W+T and Fraunhofer FHR in July 2015 are briefly described. In particular, the scenario used for the bistatic RCS measurements is presented, together with the LORA-11 passive radar system.

### 2.1. Scenario Analysis

**Figure 1:**
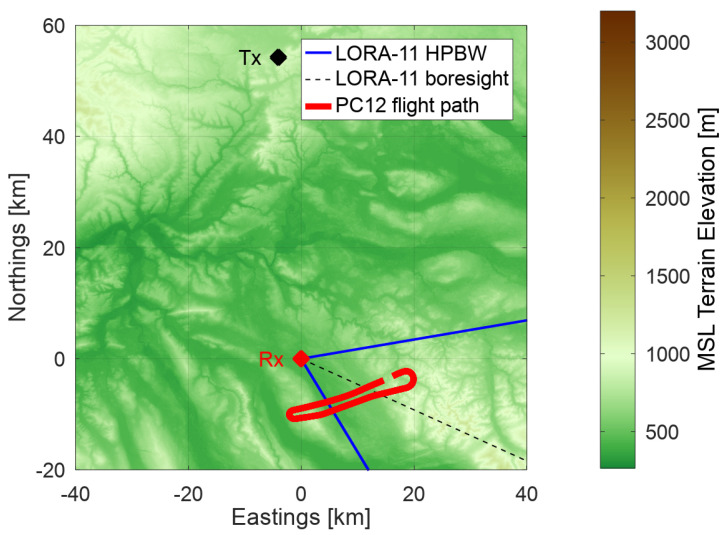
The scenario geometry measured and presented in this paper.

A map of the acquisition geometry for the measurement campaign is shown in Figure 1 with the broadcasting transmitter Donaueschingen (Tx) marked in black, the location of the LORA-11 receiver at Dübendorf airfield marked as Rx and the analyzed flight path of the PC-12 shown as a solid red line. The blue straight line indicates the single element half power beam width (HPBW) of LORA’s receiving antennas. The color coding in Figure 1 highlights the local topography.

The selected transmitter of Donaueschingen (whose main parameters are reported in Table 1) is situated at a distance of 54.4 km from the receiver location.

**Figure 2:**
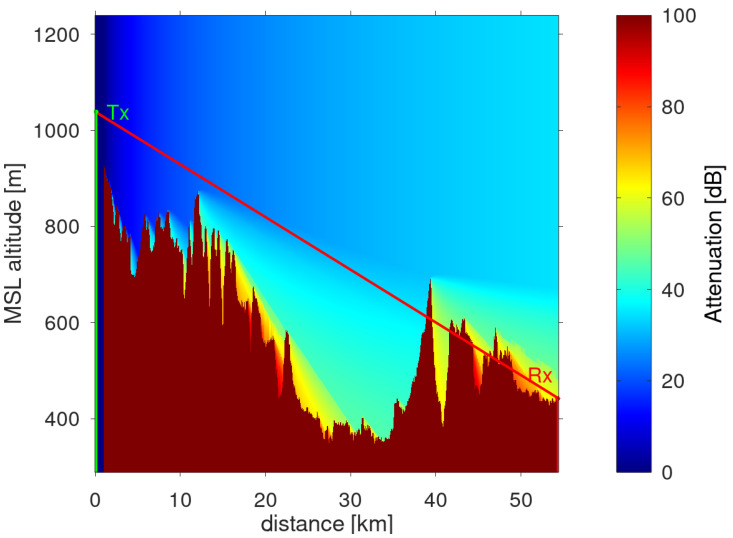
Signal attenuation prediction between transmitter (Tx) and receiver (Rx). The red line represents the hypothetical line of sight between transmitter and receiver corrected for the earth’s curvature.

To assess the transmitter signal level at the receiver location, digital terrain elevation data from the ASTER GDEM data set [22] were analyzed with FHR’s DARWIN program [23,24]. This software tool calculates propagation losses according to the recommendations in [25]. Figure 2 shows the expected signal attenuation along the propagation path between transmitter Donaueschingen (left hand side in Figure 2) and the LORA-11 receiver stationed at the airfield Dübendorf (right hand side in Figure 2). As is evident from Figure 2, the mountainous area prevents optical line of sight between the two sites. Specifically, the prediction tool estimated an additional 21.4 dB of attenuation losses with respect to optical line-of-sight propagation. Nevertheless, thanks to diffracting wave radio propagation effects, transmitted signal reception at the airfield and subsequent reconstruction was still possible.

The DARWIN program was also used to investigate whether the terrain affected free space propagation of the target echo reception, as this might directly affect the resulting bistatic RCS measurements. Onboard IMU recordings were used to analyze the point-to-point wave propagation path between the transmitter-to-target and target-to-receiver paths. This analysis showed that line-of-sight clearance of more than 0.6 of the first Fresnel zone diameter was present through the entirety of the analyzed flight trajectory on both the transmitter-to-target path and the target-to-receiver path. According to [25], diffraction losses can therefore be assumed to be negligible.

### 2.2. The LORA-11 Passive Radar System

**Figure 3:**
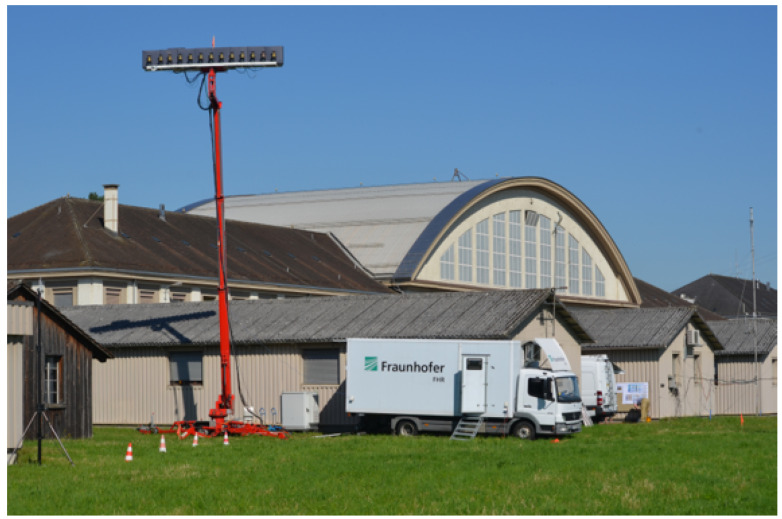
LORA-11 deployed at Dübendorf airfield during the trials.

The LORA-11 passive radar system (see Figure 3) is a software-defined, radio-based (SDR) passive radar designed to operate in UHF and VHF frequency bands, thus exploiting either DAB or DVB-T illuminators of opportunity. It consists of 12 parallel receiving channels, and it can digitize up to 32 MHz signal bandwidth simultaneously. The 12 receiving channels are synchronized with a common local oscillator (LO) disciplined via a GPS. Each receiving channel is digitized with 16 bits analog to digital converters (ADC), which ensure up to 96 dB of dynamic range. Of the 12 receiving channels, 11 are then connected to the LORA-11 antenna, which is a uniform linear array of discone elements arranged horizontally. The LORA-11 antenna enables beamforming, and it serves as a surveillance channel for target detection and localization (in azimuth). The 12th channel is connected to a dedicated antenna that is positioned in a such a way to allow for the reception of the direct signal as cleanly as possible. The antenna system is calibrated using an external reference signal of known origin. Calibration coefficients can be obtained from direct signals of the surrounding broadcasting transmitters.

## 3. Extraction of Bistatic RCS from Passive Radar Measurements

### 3.1. Range-Doppler Map Generation

A preliminary preprocessing stage synchronized the received data stream to the start of the DVB-T symbol. This also included a potential frequency offset correction due to a non-GPS locked oscillator at the transmitter and/or at the receiver side [26]. The direct signal was then retrieved by demodulating and remodulating the direct signal component from the received signal and by using the result as a reference [27]. The range compression was achieved by resorting to a reciprocal filter (RpF) [21] between the remodulated reference signal and the surveillance signal. The RpF inherently performs the range compression in a batch-wise process for each DVB-T symbol. The RpF removes any unwanted underlying information from the transmitted DVB-T signal and hence reduces the side lobes in both range and in Doppler that could potentially contaminate the target’s range-Doppler cell [28]. The range-Doppler map (RDM) was created right after the RpF, by simple Doppler processing of $M_{s}$ consecutive range compressed DVB-T symbols. The relevant signal processing parameters are listed in Table 2. The multiple available parallel receiving channels of the LORA-11 system were exploited to retrieve multiple estimates of the target RCS, which were then averaged.

**Figure 4:**
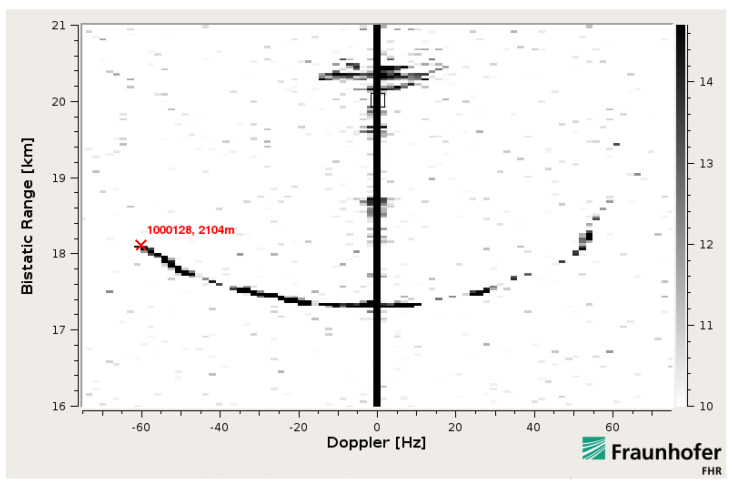
Cumulative RDM over 129 seconds. The radar track signature of the PC-12 is clearly visible in black. The vertical line at zero-Doppler corresponds to the stationary multipath. Map grayscale-scheme represents dB scale.

An exemplary cumulative RDM with highlighted target track of the PC-12 is shown in Figure 4 for a single receiving channel.

### 3.2. Target Detection and Tracking

**Figure 5:**
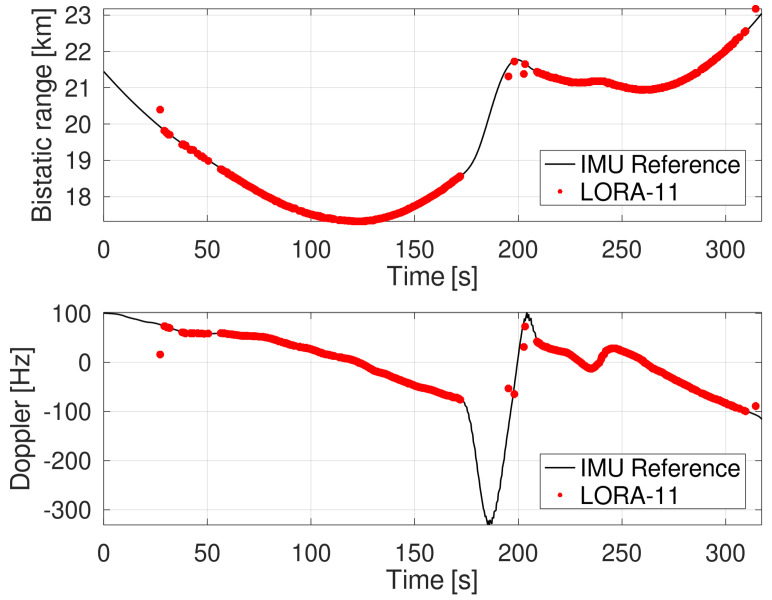
Associated tracks in bistatic range (upper plot) and bistatic Doppler (lower plot) against time. Black solid lines represent the IMU-based target information, while the red dots represent the track information derived from the LORA-11 range-Doppler maps.

The extraction of the target’s signal return levels was performed after a detection stage involving a two-dimensional cell-averaging (CA) CFAR detector with false alarm probability $P_{fa}=10^{-5}$. This step was followed by a Kalman filter-based tracking stage [29] which processed plot information in the range and Doppler domains. To ensure that target returns were correctly associated to the aircraft, target tracking was applied simultaneously to the synchronized IMU ground truth data which was transformed into the corresponding measurement domain. The resulting track heads from the reference and measurements were compared in terms of their error ellipses derived from their track’s respective covariance matrices. A preset gate determined whether the ground-truth could be associated to a given radar track. A side by side comparison of associated tracks in the bistatic range and the Doppler domain is shown in Figure 5.

**Figure 6:**
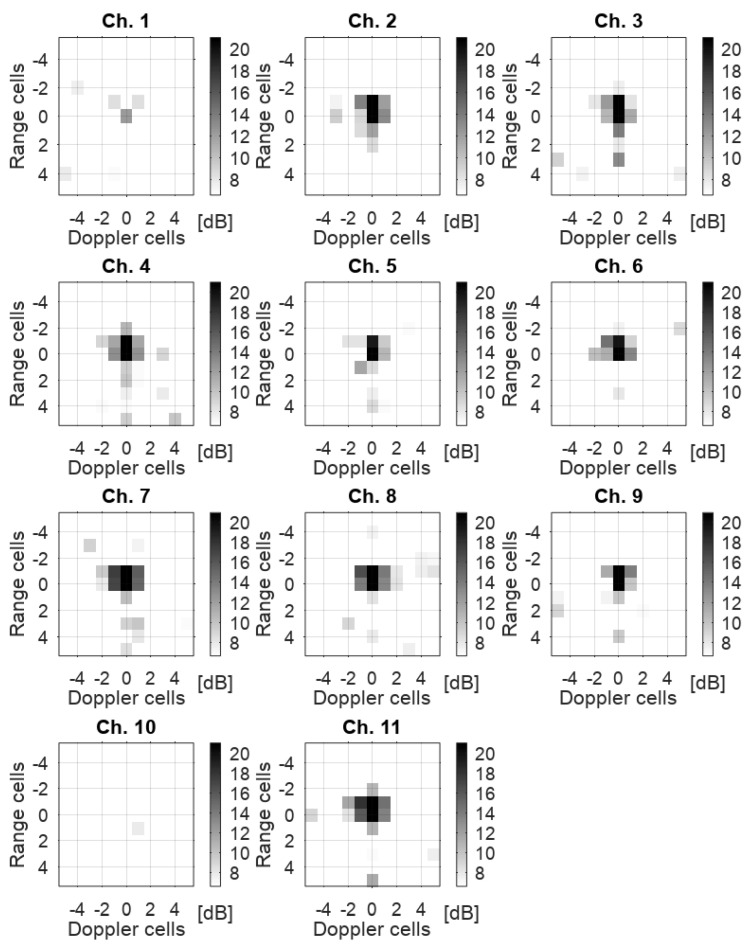
Snapshot of a single acquisition time with the detections associated to the PC-12. Range-Doppler maps cropped around the track head of the PC-12 in each of LORA-11’s 11 surveillance channels. Grid coordinates are relative to the current track head.

In terms of root-mean-square (RMS) error, the deviation in bistatic range $\Delta r_{B,RMS}$ and bistatic Doppler $\Delta f_{D,RMS}$ was 14.2 m and 3.9 Hz, respectively. As can be seen from Figure 5, the target track was lost at higher bistatic ranges due to greater path loss and during measurement times around 180 s when the aircraft was performing a turn with higher acceleration. This signal loss can be attributed to a reduction of coherent integration gain due to signal dispersal when the target migrated through multiple range and Doppler cells during a coherent processing interval. Together with signal leakage effects, this led to a spillover into multiple neighboring resolution cells. Figure 6 illustrates this effect on the RDM for each of the 11 receiving antennas of the LORA-11 system. The maps were centered around the cell associated with the target track head and cropped to highlight the energy dispersal effect.

Two comments are in order. Firstly, it is apparent that the spillover of received signal energy over multiple adjacent range/Doppler cells requires proper clustering to accumulate all the spread energy (see also Equation (2) below). Secondly, as briefly mentioned above, the measured signal power was different for each receiving antenna despite calibration. This variation can be reduced by averaging the different antennas’ measurements.

### 3.3. Extraction of Target Signal-To-Noise Ratio and Clustering

**Figure 7:**
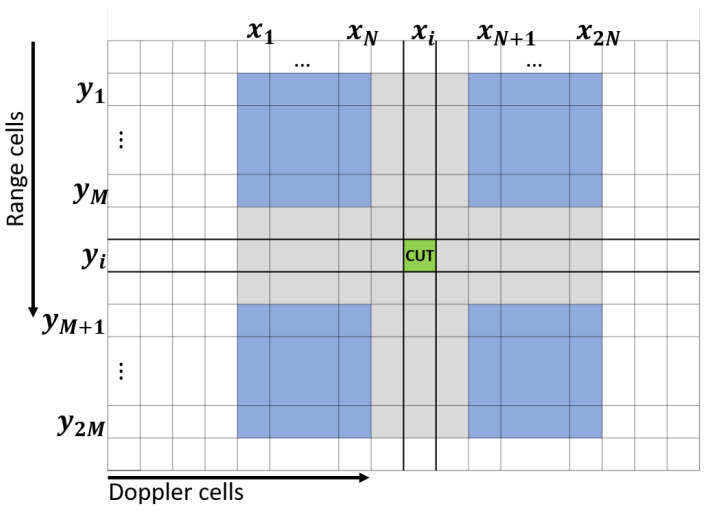
Layout of the two-dimensional sliding-reference window. Reference cells are colored blue, and guard cells are gray.

The extraction of the target signal power requires an accurate estimation of the local time dependent noise power level. This was accomplished in an adaptive way, by averaging the neighboring cells on the RDM within a two-dimensional sliding-reference window around each cell under test (CUT). A few immediate range and Doppler cells around the CUT were assigned as a guard to avoid the inclusion of the signal spillover as proposed in [30,31]. To avoid static ground clutter cells skewing the noise statistic, additional guard cells were introduced whenever an overlap of the reference window with zero Doppler cells occurred. Figure 7 shows the general layout of the sliding window.

The noise expectation value $\mu_{j}\left[x_{i},y_{i},t\right]$ for each CUT of Doppler index $x_{i}$ and range index $y_{i}$ was therefore calculated as:

(1) $$\mu_{j}\left[x_{i},y_{i},t\right]=\frac{1}{4N\cdot M}\sum_{n=1}^{2N}\sum_{m=1}^{2M}\chi_{j}\left[x_{n},y_{m},t\right],$$

with $\chi_{j}$ denoting the RDM of the channel with index *j*, generated at time stamp *t*. *N* relates to the number of Doppler cells in the reference window with indices $x_{n}$ and *M* as the number of range cells in the window with indices $y_{m}$.

To ensure that the entire energy from the target returns spread over several cells is extracted, a clustering was applied to the cells neighboring the associated track head. The total $SNR_{j}\left(t\right)$ extracted from the target in each channel was thus calculated as the sum SNR of all connected cells as,

(2) $$SNR_{j}\left(t\right)=\sum_{k=1}^{K_{c,j}\left(t\right)}\frac{{\left|\chi_{j}\left[x_{k},y_{k},t\right]\right|}^{2}}{{\left|\mu_{j}\left[x_{k},y_{k},t\right]\right|}^{2}},$$

with $K_{c,j}\left(t\right)$ as the number of cluster cells for the j-th receiving channel at time *t* with indices $x_{k}$ as the bistatic Doppler indices and $y_{k}$ as the respective range cell indices. $\mu_{j}\left[x_{k},y_{k},t\right]$ denotes the estimated noise level in the corresponding cells. As can be seen in Figure 6, SNR levels in antenna channels 1 and 10 were significantly lower because of higher noise levels present in both channels. Data from these channel was discarded in the further evaluation of the target RCS. As a result, the SNR values from the remaining $N_{A}$ = 9 receiving antennas were averaged to limit target signal fluctuations, that is:

(3) $$\bar{SNR}\left(t\right)=\frac{1}{N_{A}}\sum_{j=1}^{N_{A}}SNR_{j}\left(t\right).$$

**Figure 8:**
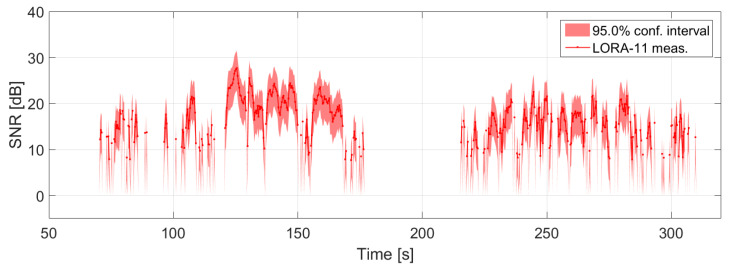
$\bar{SNR}\left(t\right)$ in dB extracted from LORA-11 data with associated confidence band.

Figure 8 reports the measured $\bar{SNR}\left(t\right)$ averaged over the nine receiving channels under consideration as a solid red line.

Since the noise values are calculated under the Gaussian assumption for the reference cells in $\chi_{j}$ outside of the ground clutter, their power amplitudes have a Rayleigh distributed probability density function. Based on this, the 95% quantiles were calculated for the estimation of $\mu_{i}$ and consequently for the SNR calculation. The confidence interval indicated by the red area in Figure 8 expresses this fluctuation.

With reference to the applied processing, a final comment is in order. We took particular care to select a scheme that was robust enough to guarantee adequate detection and that, at the same time, was able to limit unwanted SNR fluctuations over time due to varying noise level estimates (see Equations (1)–(3)) as much as possible. In particular, we found that an incoherent integration of the target returns over adjacent RDM cells (Equation (2)), as well as the averaging of the SNR values measured over the different antennas (Equation (3)), ensures a smoother estimation of the SNR over time. Please also note that a coherent integration of the target echo signal received at the different physical channels of the LORA-11 system (as sketched in Figure 6) would certainly have improved the overall SNR, but it would have introduced another potential source of error due to the need for amplitude and phase calibration of the different receiving channels. As far as the interference cancellation is concerned, we did not apply any direct signal interference removal techniques (such as ECA [32] or the CLEAN filter [33]). The reason is again related to the additional fluctuations that these approaches could generate over time (that is, from one RDM to the next one).

### 3.4. Inversion of the Bistatic Radar Equation

Let us indicate with $P_{S}\left(t\right)$ the target signal power at trajectory time *t* and with $P_{N}\left(t\right)$ the corresponding noise power. Let us also assume that the noise power $\tilde{P_{N}}\left(t\right)$ can always be estimated from the RDM by averaging in a noise-only region, and that this value is then used to normalize the entire RDM. Since a passive radar system does not have control over the transmitted signal (and consequently on the quality of the corresponding received direct signal), the noise level $P_{N}$ (or $\tilde{P_{N}}$) is also a function of the trajectory time *t*. The signal power measured in the resulting RDM at the target position $P_{tgt}\left(t\right)$ will then be the sum of the target signal power $P_{S}\left(t\right)$ plus the noise power $P_{N}\left(t\right)$ divided by the estimated noise power $\tilde{P_{N}}\left(t\right)$. That is,

(4) $$P_{tgt}\left(t\right)=\frac{P_{S}\left(t\right)+P_{N}\left(t\right)}{\tilde{P_{N}}\left(t\right)}≃SNR\left(t\right)+1,$$

assuming $\tilde{P_{N}}\left(t\right)≃P_{N}\left(t\right)$. By recalling the bistatic radar equation, the SNR over trajectory time *t* can be written as:

(5) $$SNR\left(t\right)=\frac{P_{tx}G_{tx}G_{rx}\lambda^{2}\;\sigma \left(t\right)\;N_{\mathrm{int}}\left(t\right)}{{\left(4\pi \right)}^{3}R_{tx}^{2}\left(t\right)R_{rx}^{2}\left(t\right)P_{N}\left(t\right)L},$$

where $P_{tx}$ is the transmitted signal power, $G_{tx}$ and $G_{rx}$ are respectively the transmitter and receiver gains in the directions of the target, λ is the carrier wavelength, σ(t) is the true bistatic radar cross section, $N_{\mathrm{int}}\left(t\right)$ indicates all processing gain including: (i) the coherent Doppler integration over $M_{S}$ DVB-T symbols; (ii) the compression gain of the RpF; and (iii) the incoherent integration over $K_{c,j}\left(t\right)$ RDM cells. $R_{tx}\left(t\right)$ and $R_{rx}\left(t\right)$ are the one-way ranges from the target to the transmitter and to the receiver, respectively. Finally, *L* depicts all losses at the receiver. Please note that in general the terms, $G_{tx}$ and $G_{rx}$ also depend on the trajectory time *t*.

By plugging (5) into (4) and by compensating for the time-varying range terms, the following quantity that is directly proportional to the target RCS is obtained.

(6) $$\tilde{\sigma }\left(t\right)=\gamma \cdot \sigma \left(t\right)=\left(P_{tgt}\left(t\right)-1\right)\cdot R_{tx}^{2}\left(t\right)R_{rx}^{2}\left(t\right)$$

where γ accounts for all nonvarying terms in (5).

## 4. Simulation Method

From the point of view of simulation, the required result is the bistatic RCS of the aircraft for the particular incidence and scattering angles required along the flight trajectory. If only monostatic RCS were required, it would likely be practicable to produce a lookup table and then to simply interpolate this to obtain the required RCS for the direction of interest. However, in the bistatic case, significantly more simulation time would be required, since for each incidence direction it would be necessary to determine the RCS for the full sphere around the target. Instead, we performed the simulations on an as-needed basis, explicitly performing a discrete simulation for each incidence and scattering angle pair required for the particular flight.

**Figure 9:**
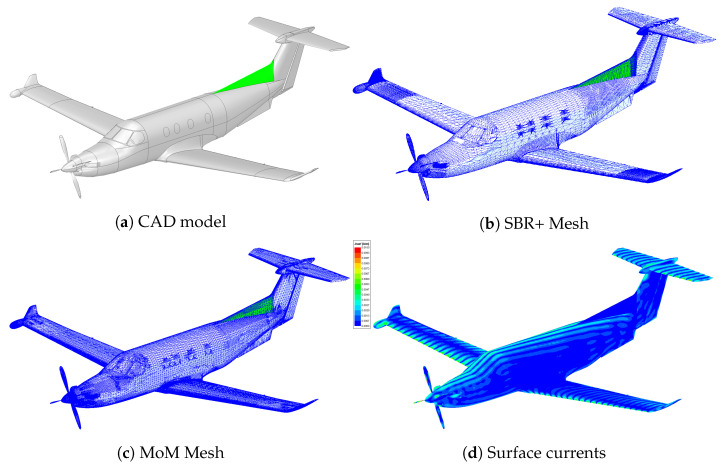
Images of the modeled aircraft showing: (**a**) the CAD model used; (**b**) the mesh at 482 MHz for the SBR+ solver; (**c**) the mesh at 482 MHz for the basic MoM solver; and (**d**) the surface currents for a sample incident plane wave arriving from the left side of the aircraft.

All of the simulations were performed in Ansys HFSS 2019 R3 (also known as version 19.5) on the 3D model of the Pilatus PC-12 aircraft shown in Figure 9a. The model was predominantly treated as perfectly electrically conducting, with only the upper fairing (shown in green) treated as glass-fiber. The fuselage was also largely treated as being a solid body, with only the gap between the rudder and main fuselage currently modeled.

All of the simulations were performed in the aircraft’s frame of reference. The incidence and scattering directions and incident polarization in that frame of reference for each point to be simulated were precalculated in Matlab based on the positions and orientations of the aircraft, transmitter and receiver. A Python script was then created to configure each individual simulation in HFSS. The aircraft’s position and attitude were determined from the GPS-aided IMU mounted onboard the aircraft. In the final version of the simulations, we assumed an ellipsoidal earth and used a four-thirds earth radius model [34] to account for atmospheric bending when determining the incidence and scattering angles, but we also consider alternatives to these approximations in Section 5.2.

The need for on-demand simulations at each point along the flight path makes the computational time required for each point significant. There is a need for a trade-off between simulation accuracy and speed. For this work, we used a full version of HFSS’s method of moments (MoM) solver (also known as the integral equation (IE) solver) at only a select set of points along the flight path that are used for error analysis. These simulations took around 9 hours per data point (at 5 frequencies). For the bulk of the flight we performed more basic MoM simulations, which differ from the full MoM simulations in that we did not allow the mesh to be iterated. These more basic simulations took around 1 h, 50 min per data point (again at 5 frequencies). We also performed even higher temporal resolution simulations using HFSS’s enhanced shooting and bouncing ray (SBR+) solver in two different modes: with physical theory of diffraction (PTD), uniform theory of diffraction (UTD) and creeping wave physics enabled (referred to later as full SBR+), and with these all disabled (referred to later as basic SBR+). The SBR+ simulations took approximately 90 seconds per data point (again at 5 frequencies), with around a 5-second saving when using the basic solver. We used the highest-quality simulations to validate the faster, lower quality simulations. All of the simulation times quoted are for a 2020 workstation with 16 cores and 384 GB of RAM.

A typical SBR+ mesh for simulations at 482 MHz and consisting of 233,241 elements is shown in Figure 9b. The equivalent single-pass MoM mesh is shown in Figure 9c, and consists of 272,277 elements. The higher number of elements is due to the need to have at least a minimum number of mesh elements per wavelength in the MoM model. An example of the typical surface currents seen for an incident plane wave, and from which the far-field electric field is computed, is shown in Figure 9d.

Once the simulated RCS, $\sigma^{\prime }\left(t\right)$, was computed for each time point *t*, we computed a term proportional to the simulated target signal power $P_{S}^{\prime }\left(t\right)$:

(7) $$P_{S}^{\prime }\left(t\right)\propto {\bar{P}}_{S}^{\prime }\left(t\right)=\frac{\sigma^{\prime }\left(t\right)}{R_{tx}^{2}\left(t\right)R_{rx}^{2}\left(t\right)}.$$

Using such a quantity for comparison rather than the simulated RCS allows for a better understanding of the system’s detection performance. In measurement, we do not have the absolute received power, so any comparisons that we make must be to the relative variations in received power and not the absolute value. As such, there is no benefit to computing the simulated absolute received power—a relative value is sufficient.

## 5. Simulation Method Uncertainties

The accuracy of the simulations comes down to three factors: how accurate the electromagnetic simulations of the model are, how well we know the geometry (ultimately, the relevant incidence and scattering directions, and the polarization), and how accurately the aircraft model represents the real aircraft. These factors will be discussed in the following section.

### 5.1. Solver Accuracy

**Figure 10:**
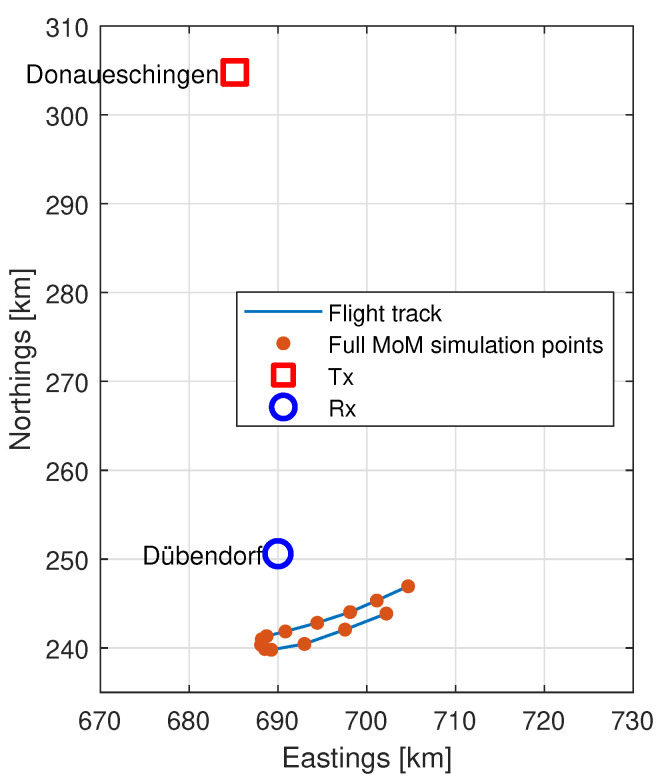
Points at which full MoM simulations have been performed.

**Figure 11:**
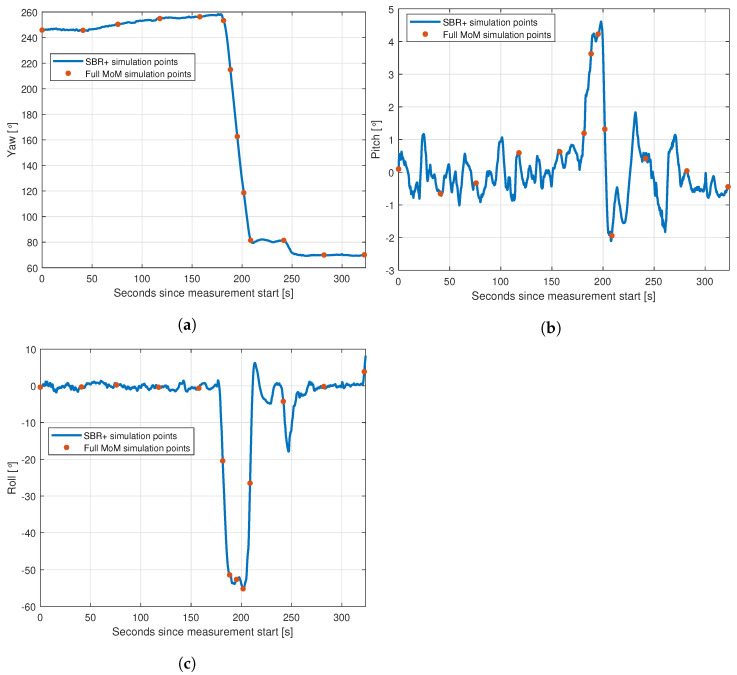
The 13 yaw, pitch and roll values used for the full MoM simulations (red dots) plotted on the full set of available values (blue lines), used for the SBR+ simulations: (**a**) yaw simulation points; (**b**) pitch simulation points; (**c**) roll simulation points.

We worked with two different solvers, each used in a more and a less accurate configuration. It is important to understand what the reduction in accuracy is with the changes in configuration. Since it would not be computationally reasonable to perform full MoM simulations of the entire flight path, we instead selected a smaller number of points along the path. We then performed full simulations on these points. We began this by selecting nine uniformly spaced data points from the entire flight period to obtain broad coverage of the full flight. This initial choice meant that most of the points were from times where the aircraft was in level flight and side-on to the transmitter and receiver, so additional points were added where the aircraft was turning and therefore where the orientation of the aircraft relative to the transmitter and receiver was changing significantly. We used the region where the magnitude of the roll exceeded 18${}^{\circ }$ as a computationally friendly proxy for this turn and selected 5 additional uniformly spaced data points from the region. One data point happened to exist in both data sets, leading to a total of 13 unique data points. These data points are shown on the flight track in Figure 10, and their yaw, pitch and roll values are shown in Figure 11.

In most cases, the full MoM simulations converged after the second iteration, with the mesh for the second step typically having approximately 354,000 elements. However, the seventh, eighth and ninth example points required three iterations to converge (in all cases only just missing the default convergence criteron on the second iteration), with the final meshes each having approximately 460,000 elements. It is likely not a coincidence that these points occurred when the aircraft was banking, was pitched up, and had its tail to the transmitter and receiver, as opposed to the side-on illumination and near level flight of the other sample points, but we do not have enough data to be certain about which of these factors are significant, or how general this behavior is.

When comparing each of the five simulated frequencies independently, the largest difference between the full and basic MoM simulations was 1.72 dB, the mean difference 0.44 dB and the median difference 0.31 dB. If the means of the RCSs across the five simulated frequencies are computed and then the differences of these mean values are taken, the resultant maximum, mean and median differences are 1.38 dB, 0.36 dB and 0.27 dB. There are not a sufficient number of data points here to draw truly general conclusions about the effect of the measurement geometry on the scale of the error in our basic MoM solver method, but for the purposes of our measured flight, the errors are small, particularly when compared with the 34 dB range of simulated RCS values.

**Figure 12:**
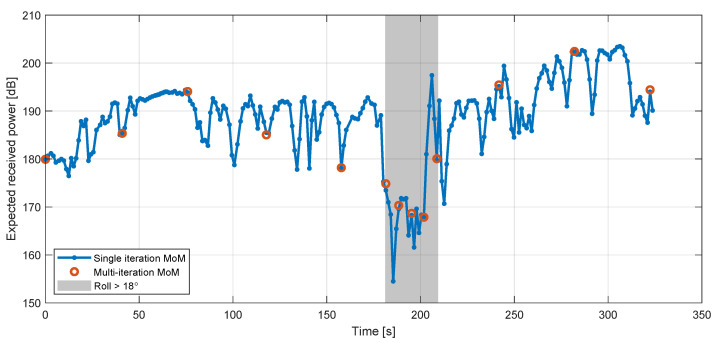
Comparison between the frequency-mean simulated received-power values (Equation (7)) computed with the single- (basic) and the multi-iteration (full) MoM methods. The grey box indicates the times during which the roll exceeded 18${}^{\circ }$, and is intended as an approximate indication of the times where the aircraft was turning.

The impact of the errors can be seen in Figure 12, which shows the means of the five-frequency received-power values (${\bar{P}}_{S}^{\prime }\left(t\right)$, Equation (7)) computed with both the basic (single iteration) and full (multi-iteration) MoM simulation methods.

**Figure 13:**
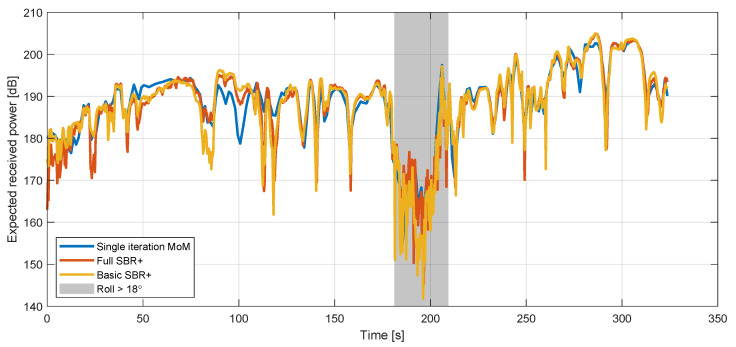
Comparison between the frequency-mean simulated received-power values (Equation (7)) computed with the single-iteration MoM method and the two different SBR+ methods. The grey box indicates the times during which the roll exceeded 18${}^{\circ }$, and is intended as an approximate indication of the times where the aircraft was turning.

**Figure 14:**
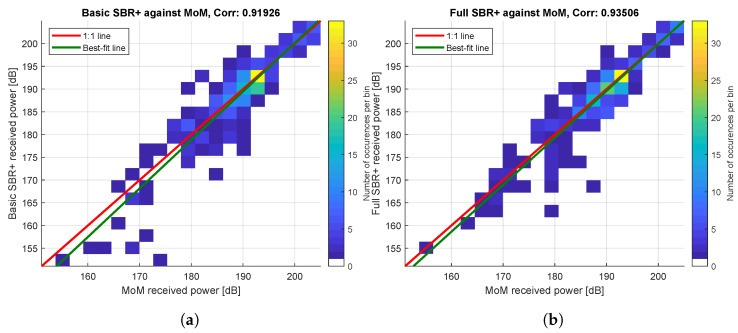
Comparisons between the single-iteration MoM simulation received-power values and the basic and full SBR+ simulation received-power values: (**a**) Basic SBR+; (**b**) Full SBR+.

Similar comparisons between these full MoM simulation points and the full and basic SBR+ simulations yield mean errors of 3.2 and 5.8 dB, respectively. In both cases this is driven by a single extreme value for one data point, but in the basic SBR+ case, there are nevertheless three data points at which the error exceeds 10 dB. However, for the SBR+ simulations, we can obtain a more robust estimate of the error in the SBR+ method from comparisons with the basic MoM values at their 242 common simulation points, which yields slightly lower mean differences of 2.1 and 2.5 dB, respectively. More generally, the correlation between these values is excellent, as shown in Figure 13 and Figure 14. Specifically, Figure 14 shows two two-dimensional comparison histograms between basic SBR+ and basic MoM (left-hand side) and between full SBR+ and basic MoM (right-hand side), respectively. These two histograms are overlaid with solid red lines indicating the 1:1 matching of the simulated received-power values between the two solvers (corresponding to full correlation). The green lines indicate the best linear fit to the data. It is apparent that the data fitting lines in green offer a good match to the perfect correlation lines in red for both comparisons. We conclude that, for the performed comparison, the full SBR+ simulation performs better than the basic SBR+ simulation, but that the difference is small.

As a result, we believe that the error introduced by using the basic MoM simulation method rather than the full MoM method is very small, typically at around the 0.36 dB level. The SBR+ simulation method introduces a significantly larger error, but it is still small compared to the overall range of values considered. We therefore rely mainly on the larger number of simulations possible with the SBR+ method for much of the remainder of the error analysis.

### 5.2. Angular Error

Errors in the assumed incidence and scattering angles can arise from both the approximations made in computing them and from uncertainty in the aircraft’s position and attitude. We assume that our uncertainty in the position of the transmitter and receiver is negligible, since more accurate measurement methods are available for such stationary objects, and in any case, the positions are fixed for the entire flight. In this subsection, we consider first the geometric approximations made, the atmospheric approximations made, the uncertainty in the aircraft’s position and finally, the effects of integrating over time periods in which the aircraft moves. The various angular errors are then related to errors in the actual RCS.

#### 5.2.1. Geometric Approximations

**Figure 15:**
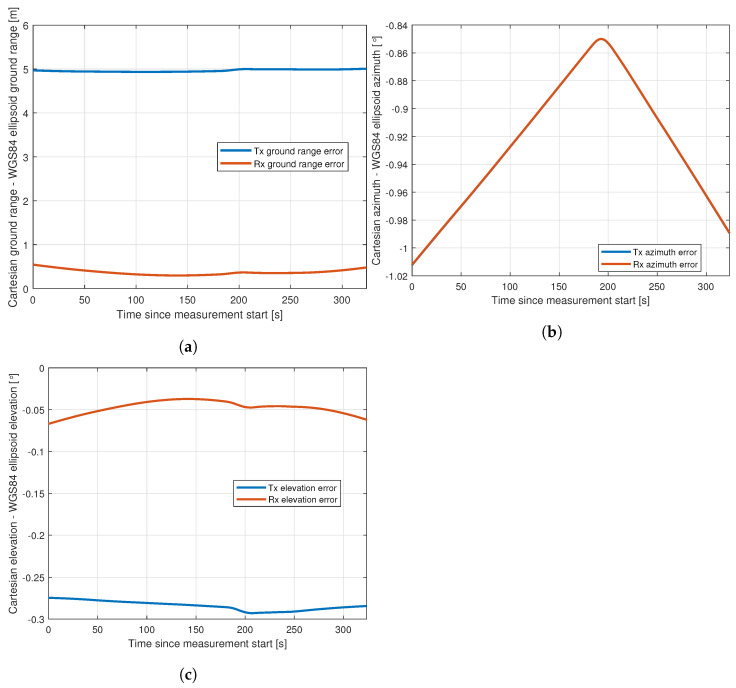
The error in ground range, azimuth and elevation between the transmitter and aircraft and receiver and aircraft resulting from using a Cartesian approximation along the ground range compared with an ellipsoidal earth approximation: (**a**) ground range; (**b**) azimuth; (**c**) Elevation.

Over shorter ranges, it is always simpler and often sufficient to use a local coordinate system that can be treated as Cartesian for the purpose of calculating range and incidence and scattering angles. Whether this is sufficient in any particular case will depend on the ranges and directions involved, the location on the globe, the local coordinate system used, and the sensitivity of the simulations to these errors. We therefore restrict our analysis to our particular case, where we deal with objects in and near Switzerland and ranges of up to 65 km (transmitter to target range). We use the CH1903 (Swiss grid) coordinate system as our approximately-Cartesian coordinate system, using the conversion scheme in [35] to convert the WGS84 position and altitude values reported by the aircraft’s IMU. For a more exact method, we calculate the great-circle azimuth and range angles between the transmitter/receiver and the target position on the surface of the WGS84 ellipsoid. The resultant range and azimuthal incidence angle errors are shown in Figure 15 along our flight track.

The range error can be up to around 5 m in our case, but since this is over a range of many kilometers, and since, from a simulation point of view, it affects only the scaling of the reflected signal from the aircraft, not the aircraft orientation, it is highly unlikely to be a significant factor.

The mean azimuthal error is similar for both the transmitter and receiver, at approximately 0.93${}^{\circ }$, which is potentially significant. This error would be less if we were operating closer to the center of the coordinate system, but it is clear that it cannot be assumed that a Cartesian approximation is sufficient.

Similarly, the incidence elevation angle can be computed either from a simple Cartesian calculation in the Swiss grid, or less approximately by computing the distances on a locally spherical (with the radius of the WGS84 ellipsoid at that point) earth. The resultant errors of these two methods are shown in Figure 15c along our flight track. The difference in our case comes close to 0.3${}^{\circ }$, but we note that this is only around one-third of the error from the Cartesian azimuth approximation.

#### 5.2.2. Atmospheric Approximations

An additional source of error in our calculations comes from atmospheric bending. This can be accounted for in a number of ways, including, in order of increasing complexity: neglecting it (as with the Cartesian and spherical earth approximations in the preceding section), using a four-third earth-radius model [34], using a standard-atmosphere model appropriate for the time and place, using a measured/modeled vertical structure for that time and place, or using a full 3D measured/modeled atmosphere for the area. The last of these is beyond the scope of this paper, but we can form an idea of the extent of the error introduced from the first four.

For this, we make use of the temperature, pressure and humidity profiles for standard atmospheres given by the ITU in [36], calculating the resultant air refractive index [37]. We then use this to calculate the propagation of the signal through an assumed spherically stratified atmosphere (standard method, found in, e.g., Appendix A of [34]) to determine the propagation path through the atmosphere to the aircraft. The key parameters that we determine are the incidence and scattering elevation angles at the aircraft. We assume for this error analysis that the transmitter and receiver antenna gains are constant and that the small shift in range due to atmospheric effects is negligible. Were the last of these not the case, the same method could also be used to take this into account.

In addition, MeteoSwiss provided us with 7 km resolution COSMO-7 [38] temperature, pressure and humidity reanalysis data at 10-min increments for the measurement time and area, interpolated to a 100 m vertical resolution. We averaged these data at each altitude and used the same method as for the standard atmospheres to calculate the resultant path through this atmosphere. We use this as a baseline against which to compare the other models.

**Figure 16:**
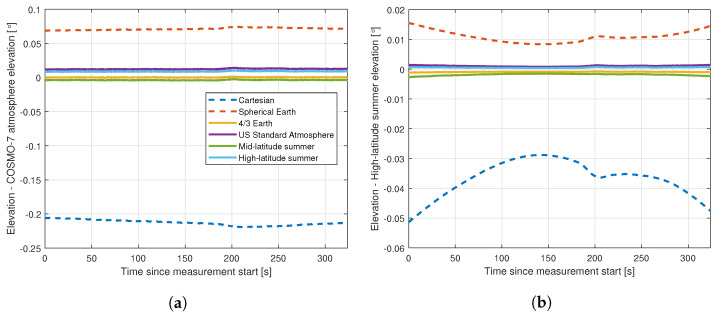
The error in the elevation angles resulting from various methods of accounting for atmospheric bending, compared with a COSMO-7 reanalysis of the atmosphere at the time of the measurements: (**a**) transmitter; (**b**) receiver.

We show the results of this comparison along our flight track in Figure 16. Of the standard atmospheres, our latitude (46–47${}^{\circ }$ N) puts us just into the ’upper latitude’ model (threshold 45${}^{\circ }$ N, and since the measurements were made in July, summer is the more appropriate version of this standard atmosphere to use, but we also show the US standard atmosphere and the mid-latitude summer atmospheres for comparison. In practice, in our specific case, the four-thirds earth model atmosphere produces angles very close to those from the COSMO model, most notably for the transmitter angle.

From this we conclude that the error associated with the no-atmosphere approximations is significantly larger than the difference between any of the with-atmosphere results. The maximum variation of the apparent elevation angle of the transmitter at the location of the aircraft with different atmospheres is 0.034${}^{\circ }$, and 0.008${}^{\circ }$ for the closer receiver. The maximum difference between the four-thirds earth model and the most plausible high-latitude summer model is 0.009${}^{\circ }$ for the transmitter and 0.002${}^{\circ }$ for the receiver, i.e., far less than the variation between the different atmospheric models. In practice, for our case, we find that the four thirds earth model atmosphere is actually closest to our most sophisticated model, so we use this for our calculations. However, this is not a general result; if working under different conditions it would be necessary to revisit this assumption.

#### 5.2.3. Attitude and Position Errors

From the orientation information provided by the aircraft’s IMU, we expect an error of 0.1${}^{\circ }$ for pitch and roll and 0.2${}^{\circ }$ for yaw [39]. We also expect position errors of 2 m in eastings and northings, and 3 m in height. All of these factors introduce uncertainty into the calculated incidence and scattering azimuth and elevation angles. If we assume that the errors are normally distributed around the true value and that the error values from the IMU manual correspond to the standard deviations, then, for our particular geometry, the median expected uncertainty of the combination on the incidence and scattering angles in the aircraft’s coordinate system are 0.14${}^{\circ }$ in azimuth and 0.07${}^{\circ }$ in elevation. In our case, these angular errors arise almost entirely from the attitude uncertainties rather than the position uncertainties, but at very short ranges, they would become significant. These uncertainties are potentially significant, and are difficult to avoid without using a more accurate IMU.

#### 5.2.4. Changing Position over Time

Finally, since the measurements are integrated over 573 ms and the aircraft moves in that time, the measurement is really of the integrated RCS across these angles and orientations. Since the IMU update rate (20 Hz) is far higher than the integration time, we know the location and aircraft at 11–12 points for each measurement time. We can therefore calculate the variation of the incidence and scattering angles over these times for our flight. The median variation in the azimuth angle of the transmitter is 0.12${}^{\circ }$ and to the receiver of $0.023^{\circ }$. The equivalent median elevation variations are 0.00031${}^{\circ }$ and 0.016${}^{\circ }$, respectively.

#### 5.2.5. Effect on RCS

To determine which, if any, of these effects are significant, it is important to determine the impact of azimuth and elevation errors on the simulated RCS along our flight path. With the four-thirds earth radius approximation, we have simulated the RCS at each of the 13 error points (Figure 10), whilst adding offsets to the incidence and scattering angles of −0.15${}^{\circ }$ to 0.15${}^{\circ }$ in 0.02${}^{\circ }$ increments. The incidence and scattering azimuth and elevation angles were varied separately, in all cases with the other three values fixed at their central values, leading to a total of 832 different angles being simulated, each at 5 frequencies. The simulations were performed using the full SBR+ solver.

**Figure 17:**
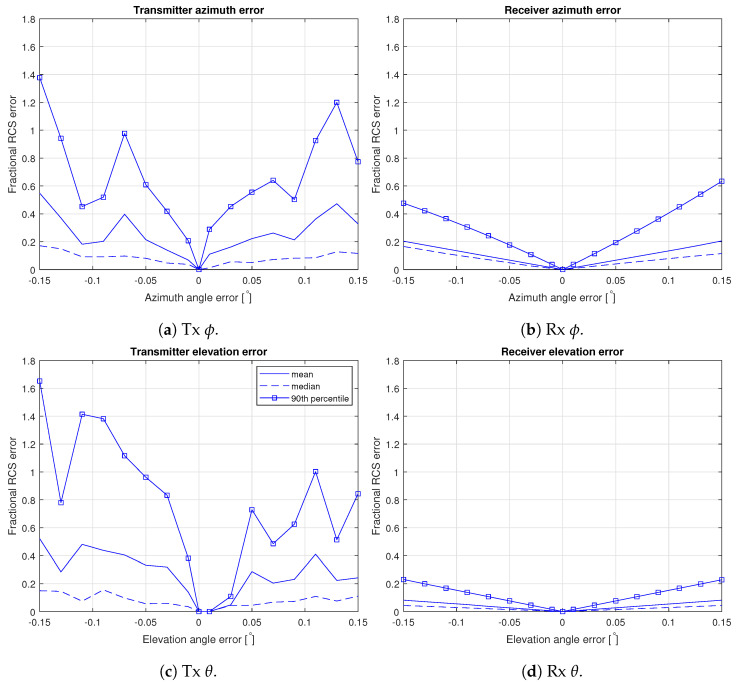
RCS errors as functions of increasing angular errors for each of the incidence angles, calculated separately.

From these bistatic RCS values, the mean, median and 90th percentile of the fractional RCS error (relative to the central RCS value) were calculated and are shown in Figure 17. From these it is quite clear that for our geometries the error introduced by changing the angle to the transmitter is more significant than that introduced by changing the angle to the receiver.

These results, combined with the fact that the error in elevation angle from the transmitter due to the four-thirds earth radius model is so small (median 0.00023${}^{\circ }$, max 0.0013${}^{\circ }$), allow us to conclude that the error introduced by the atmospheric assumption is negligible.

The same is not, however, true for the attitude and position errors. From Figure 10, we can estimate that the median fractional errors introduced in the calculated RCS values are approximately 0.14 for the transmitter azimuth, 0.13 for the receiver azimuth, 0.085 for the transmitter elevation and 0.02 for the receiver elevation. A quadrature combination of these yields an overall error estimate of 0.21. Note that this is really only a measure of the typical error. If we take instead the 90th percentile, the resultant overall error estimate is actually 1.48.

**Figure 18:**
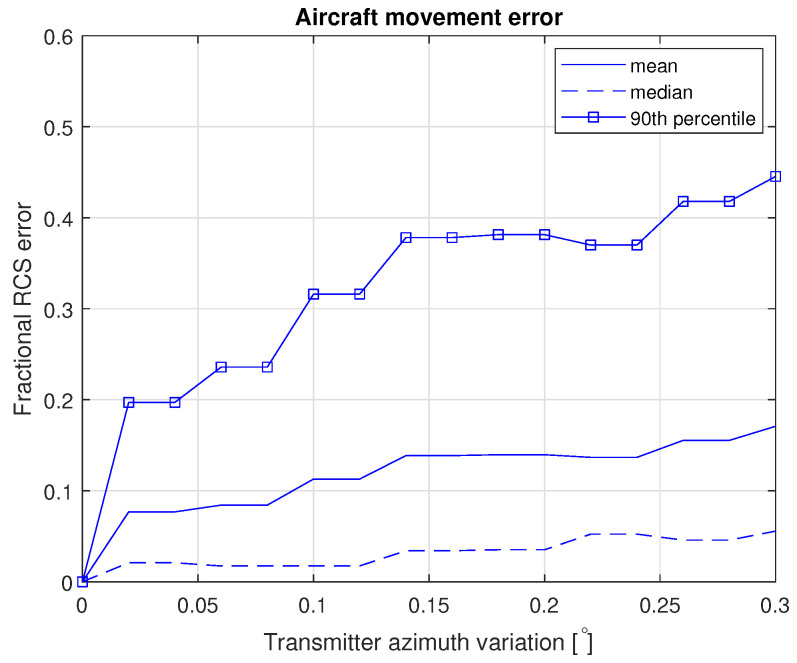
Angular error in position as a function of integration time.

This still leaves the error due to neglecting the aircraft’s movement during the measurement integration time. This error will largely be in azimuth, and we further know that the dominant effect is the error to the transmitter for our flight path. From the same RCS dataset used for the earlier error analysis, we calculated the fractional error in the RCS as a function of the range of the integration angle (relative to the center-point RCS of each simulation), which is shown in Figure 18. Finally, since the measurements are integrated over 573 ms and the aircraft moves in that time, the measurement is really the integrated RCS across these angles and orientations. Since the IMU update rate is far higher than the integration time, we know the location and aircraft at 11–12 points for each measurement time. We can therefore calculate the variation of the incidence and scattering angles over these times for our flight. The median variation in the azimuth angle of the transmitter is 0.12${}^{\circ }$ and to the receiver of $0.023^{\circ }$. The equivalent median elevation variations are 0.00031${}^{\circ }$ and 0.016${}^{\circ }$, respectively. Treating the 0.12${}^{\circ }$ as by far the dominant effect, we can then estimate that this leads to a 90th percentile fractional uncertainty in the RCS of around ±0.31.

### 5.3. Frequency Error

**Figure 19:**
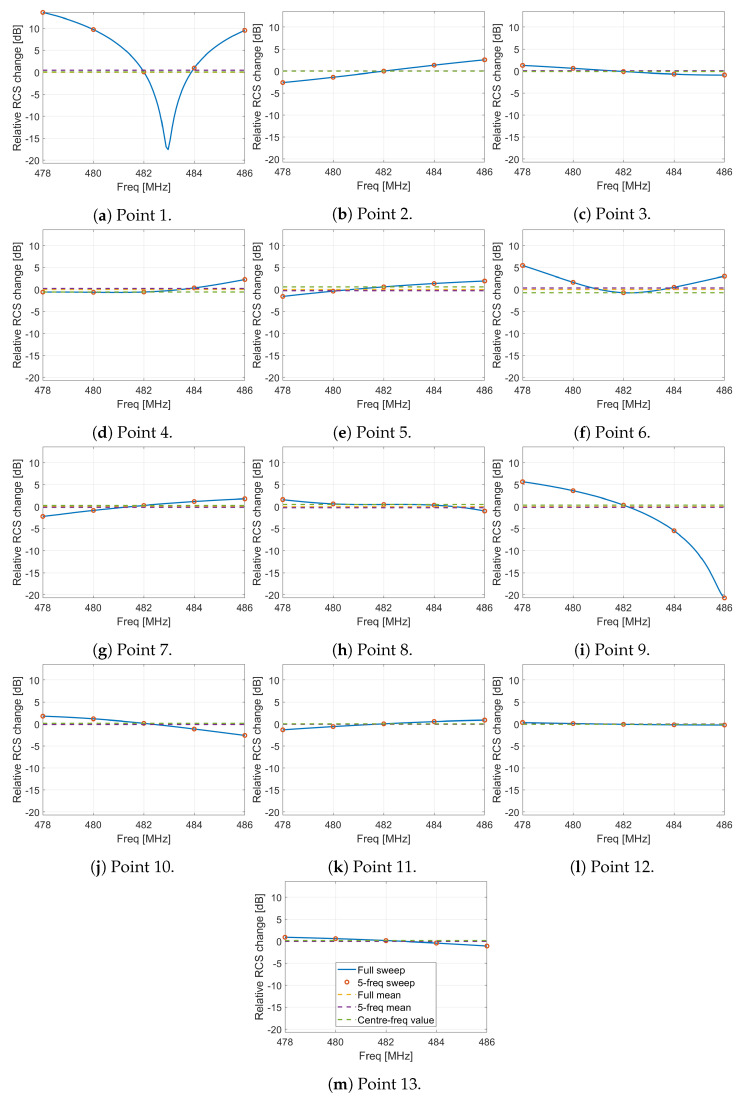
The variation in the simulated RCS with frequency at the 13 error points.

An additional issue for the simulations as compared with the measurements is that the simulations are performed at single frequencies in the frequency domain, whereas the real measurements are spread across the entire measurement bandwidth (and, indeed, at lower power levels beyond the nominal measurement bandwidth). To somewhat compensate for this, we simulate the RCS at five discrete frequencies across the measurement bandwidth and average the result. However, it is important to understand how well this improves the estimate over a single frequency and the residual uncertainty remaining. To address this, we performed SBR+ simulations at the 13 full MoM simulation points with a much finer frequency resolution (101 points across the band). In Figure 19 we show the variation in the RCS across the band, along with the RCS values obtained from just the central frequency and from averaging the five frequencies used in our other simulations.

At these 13 points, the maximum error arising from simulating only 5 frequencies rather than 101 is 0.48 dB, and the mean error is 0.16 dB. If we were to simulate only a single frequency instead (and, in doing so, substantially reduce simulation time), the maximum error is 0.73 dB and the mean 0.29 dB. In these cases, using five frequencies rather than the single center frequency approximately halves the error relative to the full sweep.

However, this is not the key issue addressed by the five-frequency sweep. The larger concern is that, as can be seen in points one and nine, there are some places where there is a null at a certain frequency. In these cases, if the single frequency point were very near the null, the error would be huge. For example, in the case of point one, the RCS value across this small frequency range varies by 31 dB. These nulls result from near-perfect destructive interference of the scattered signal in one particular direction at one particular frequency. Experience with simulations of other objects shows that these nulls are generally less deep when a MoM solver is used than with the SBR solver used here, but they occur with both. Simulating at more than one frequency significantly reduces the error in such a case. It is principally for this reason that we perform all of our simulations at five frequencies rather than just one.

### 5.4. Copper Model Comparison

**Figure 20:**
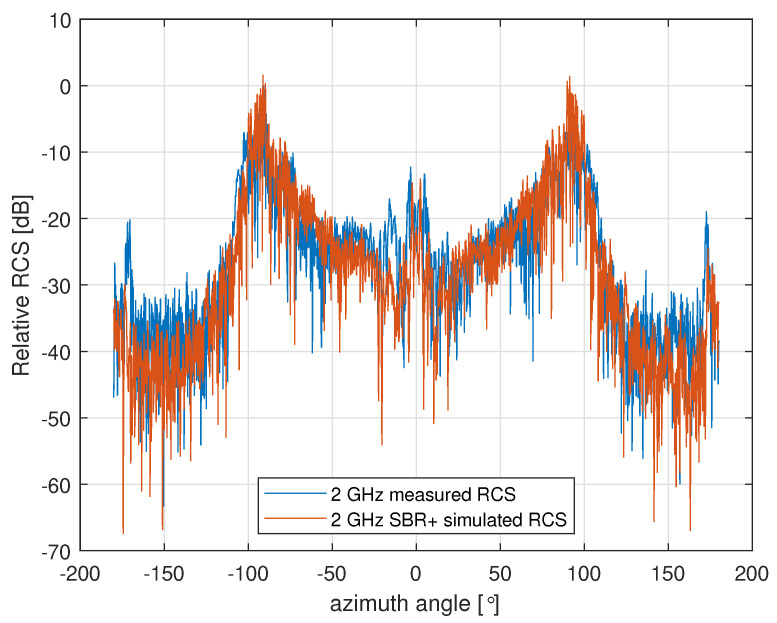
Comparison between monostatic RCS measurements of a copper scale model of the PC-12 and SBR+ simulations. MoM simulations were performed at a smaller number of incidence angles using the MoM solver, and they show similar agreement (not shown). RCS is relative to the peak measured RCS.

It is important to validate how well our simulation results compare with real measurement values in a highly controlled setting. Ideally, this would be performed on the actual aircraft, at the frequencies of interest, and for the full bistatic geometry of interest. Unfortunately, however, it was not possible for us to perform such measurements. Instead, we performed monostatic anechoic chamber measurements of a 1:8 scale copper model of the PC-12 over the range of 8–16 GHz in 10 MHz increments, and with a 0.2${}^{\circ }$ angular resolution. We performed equivalent SBR+ simulations of an all-copper version of the CAD model shown in Figure 9a from 1–2 GHz (equivalent to 8–16 GHz on the scale model). An example of direct comparison between the simulated and measured RCS values at 2 GHz are shown in Figure 20, where generally good agreement can be seen.

These comparisons do not guarantee that lower frequency bistatic RCS measurements of the real aircraft will compare well with the simulations, but they do point toward the simulations being a reasonably accurate representation of reality.

## 6. Simulation and Measurement Comparisons

**Figure 21:**
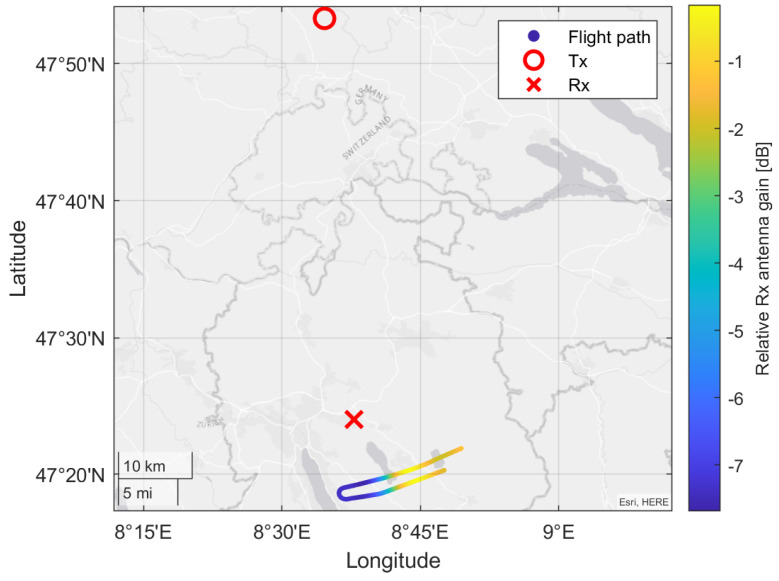
The variation of relative receiver antenna gain along the flight path, alongside the transmitter and receiver locations. Note that there is a significant reduction in receiver gain around where the aircraft turns.

To fully compare the simulation and measurement results, it is necessary to correct for the varying receiver antenna gain along the flight path. The required correction is computed from the antenna pattern, and its value is shown along the flight path in Figure 21. For our comparison, we apply this weighting to the simulated received-power values.

**Figure 22:**
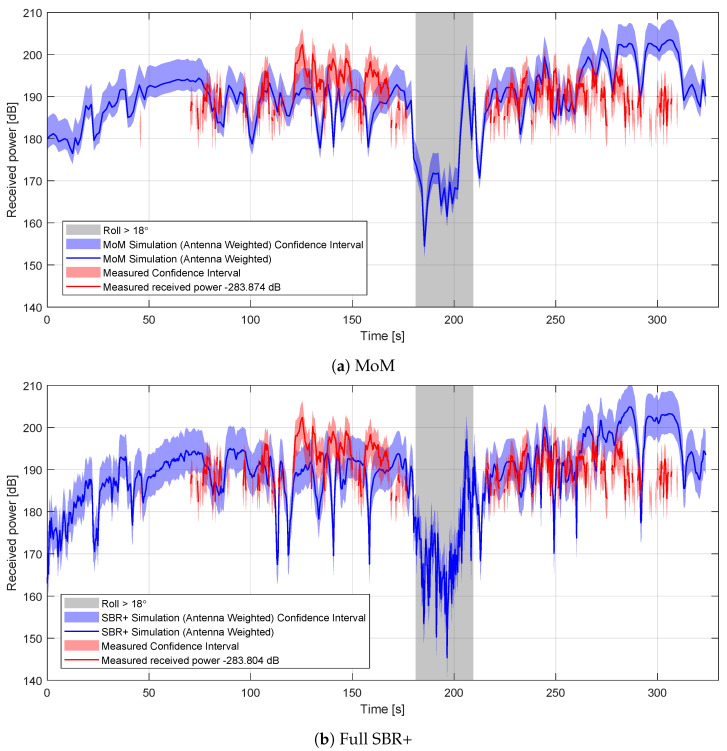
Comparisons between the simulated and measured received-power values (Equation (7)) over the measurement period. Since the absolute measured received-power value is not known, an offset has been applied to the measured data to minimize the mean difference between the simulated and measured power values in all cases where measurement data exists. The values of these offsets are shown in the figure legends and are slightly different in the MoM and SBR+ cases.

Time-series comparisons between the simulated and measured received-power values are shown in Figure 22a for the MoM simulations and Figure 22b for the full SBR+ simulations. Since absolute received-power values are not available from the radar system, we apply an estimated correction to the measured values determined from the mean of the difference between the simulated and measured received-power values for all cases where measured received-power values are available. This means that the comparison is restricted to variations in the received power and not the absolute received-power values. The offsets are applied to the measured data and are given in the figure legends.

The confidence intervals are estimated from the fractional difference between the highest quality MoM estimation and the MoM/SBR+ estimations used, relative to the MoM/SBR+ estimation used. Since the errors are generally not symmetric around the MoM/SBR+ estimations used, we consider over- and under-estimation separately. We used the 90th percentile of the error as our estimation for the error in each component. This is inherently quite impse, owing to the small number of data points available, but it provides an approximate guide to where the measured signal is plausibly expected to be. From the error analysis in Section 4, the dominant error sources are the azimuthal angle variation with integration time, the five-frequency approximation, the IMU attitude data and the simplifications in the model. The two-sided integration time-fractional RCS errors are +0.46, −0.10 for an azimuth variation of 0.12${}^{\circ }$. The two-sided frequency errors are +0.10, −0.061. Estimates are from the SBR+ solver. It is likely that this is a slight over-estimation for the MoM solver, but since the equivalent MoM simulations would be too time-consuming to perform, we work with the SBR values. The two-sided IMU position errors are +1.97, −0.40. Finally, the two-sided model errors for MoM are +0.28, −0.1, and for the full SBR+ simulations, they are +2.44, −0.63 (determined from the comparison between the basic MoM and the full SBR+ simulations, since the larger number of data points makes the estimate more robust). The combined fractional errors are therefore +2.04/−0.43 for the MoM solutions, and +3.17/−0.76 for the full SBR+ solutions.

**Figure 23:**
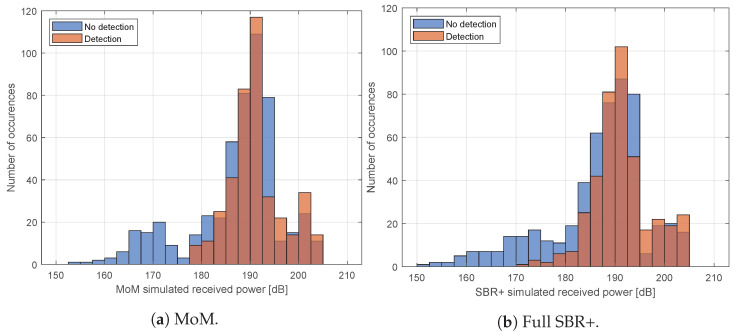
The simulated received-power values corresponding to times where the aircraft is not detected in measurement (blue), and where it is detected in measurement (orange). A high simulated received-power value does not necessarily guarantee detection (although it makes it slightly more probable), but a low simulated received-power value does guarantee that the aircraft will not be detected.

From these figures, it appears that there are some areas where the characteristics of the curves in the simulated and measured received values broadly agree in terms of the locations and relative amplitudes of peaks and troughs. However, there are other areas where this is clearly not the case. The simulations do, however, show some predictive power in being able to show where the aircraft cannot be detected in measurement: specifically when the aircraft is rolling, the simulated received-power values are particularly low, and the aircraft fails to be detected in measurement. This can be seen more clearly in Figure 23 where we show histograms of the received power for both the detection and no-detection cases.

## 7. Discussion

We performed a set of DVB-T passive radar measurements on the flight of a Pilatus PC-12 aircraft. We were able to locate the aircraft during its flight and estimate the received power. After correcting for range drop-off, we extracted a parameter proportional to the bistatic RCS of the target. The RCS of the aircraft was simulated along the same flight path, using both a MoM and an SBR+ solver to determine the expected RCS. A detailed investigation of the requirements for these simulations was performed, relating to both the solver type and configuration and to the way in which the simulations were set up. This investigation considered only a single flight of a single aircraft with a single transmitter and receiver, and so the results largely cannot be considered to be truly general. Nevertheless, there are a number of points that will apply in many cases, and they should be considered carefully in any future simulation attempts.

In particular, we found that simulating only a single frequency instead of multiple frequencies across the measurement bandwidth introduced a mean error of only 0.29 dB, but saw clear evidence that in some circumstances it could lead to enormous errors in the estimated RCS. In one extreme case, we saw RCS variation of 31 dB within the frequency band with the SBR+ solver. A similar (but likely smaller) effect would also be observed with a MoM solver. Simulating at least a small number of frequency values across the measurement bandwidth will drastically reduce the error in the simulated RCS in such edge cases and is therefore strongly recommended.

We also found that for our geometry, the bistatic RCS had a very strong dependence on the incidence angle, most notably on the elevation angle of the transmitter. To ensure that the fractional error in the RCS was less than 0.5 in 90% of cases it was necessary that the incidence elevation error be less than −0.015${}^{\circ }$/+0.023${}^{\circ }$—a very challenging level of precision to achieve. However, if only the mean fractional error is required to be less than 0.5, the incidence elevation error can be far larger: on the order 0.15${}^{\circ }$ or greater. In addition, the median fractional error in the RCS is less than 0.5, out to even greater angular errors (beyond the range simulated). The incidence azimuth angle was found to be almost as important, but the receiver azimuth and elevation angles are less so. A high level of precision in the scenario geometry is therefore required for comparisons between simulation and measurement.

If the aircraft’s motion is large enough within the measurement integration period to lead to large incidence angular variation, then it would become necessary to perform simulations at several steps along the integration path. In our case, where the azimuth angle to the transmitter varied by 0.12${}^{\circ }$ on average during each integration period, the 90th percentile fractional error in the RCS was approximately ±0.31, but in the general case, this will vary with integration time, distance to the transmitter/receiver and target velocity and should be estimated based on the expected scenario.

Other factors that would affect the calculation of incidence angles were also considered. In terms of azimuthal angle, it was important that we work with an ellipsoidal earth—simply treating the Swiss grid coordinate system as Cartesian, was not sufficient (although this issue could also be fixed with the use of the meridian convergence, e.g., [40]). The size of the error will be very much dependent on the local deviation between true north and grid north, but in our case, it was nearly 1${}^{\circ }$.

For elevation angle at the ranges we considered (up to around 65 km), atmospheric effects must be taken into account: failure to do so in our case would result in an error on the order of 0.07${}^{\circ }$ when a spherical earth model is assumed, and on the order of 0.2${}^{\circ }$ with a Cartesian approximation. Both of these errors would be significant given the sensitivity of the RCS to small changes in elevation angle. On the other hand, for our application, there was little difference in the calculated elevation angles between the true (reanalysis) atmosphere for the measurement time period, the various applicable ITU standard atmospheres and the four-thirds earth radius approximation. Given its computational simplicity, we therefore recommend the use of the four-thirds earth radius approximation except in cases where the real atmosphere is expected to deviate substantially from the standard atmospheres.

For comparing simulation results against measurements, the uncertainty in the aircraft attitude information can also be significant and is a significant source of potential error in our case. Care should be taken to choose a sufficiently accurate IMU for such measurements.

The choice of solver is highly significant in terms of required computation time, but of course it also has some impact on accuracy. Using only the initial mesh for HFSS’s MoM solver introduced a mean error of only 0.36 dB whilst requiring only one-fifth of the time to simulate. The approximate SBR+ solver we investigated reduced the required time only 0.27% of the time of the iterative-mesh MoM solver, but at the cost of a mean error of 3.2 dB (when PTD, UTD and creeping wave physics were enabled). This is a significantly large error, but it is more than adequate to resolve the variability in the RCS. Moreover the dramatic decrease in simulation time (and memory requirement) make it suitable for use in a wider array of situations. If, for example, it was desired to generate a large library of example targets and trajectories, the speed of an SBR-like solver would be an enormous advantage. We note that, whilst the simulations presented here were all performed in Ansys HFSS, similar solvers are available in competitors’ products (FEKO, for example) and similar results should be obtainable with such products.

A final important consideration is how well the model simulated represents the real aircraft. We were able to confirm that our model compares well with anechoic chamber measurements of a scale copper model, which gives us some confidence. However, we cannot say with certainty whether the copper model RCS approximates the real aircraft’s RCS well since we do not have comparable measurements of the real aircraft.

We find significant differences between the passive radar-simulated received-power levels and measurement, beyond that which can be explained by the known limitations of either our simulations or our measurements. In some areas of the comparison, the characteristics of the simulated and measured curves broadly agree, but there are other areas where this is clearly not the case. It is unlikely that other targets in the scene are a source of the discrepancies. Any such targets should appear at most only fleetingly in the same range-Doppler cell as the aircraft and would therefore have only a very brief impact on the measurement results. The source of the disagreement must come from at least one of three sources: either substantial differences between the simulated model and the real aircraft, unaccounted for propagation effects, or problems with the measurements or measurement processing. The SNR is generally quite low in the measurements performed, and the processing chain is intended primarily for detection rather than estimation, so it is possible that there is uncertainty in the measurements beyond that which is currently assumed.

Another possible source of the discrepancy is in the propagation of the signal between the transmitter and the aircraft due to interaction with the terrain/features on the ground. We know that we have a clear direct line of sight to the aircraft, but it is at least possible that interactions elsewhere in the main lobe result in fluctuation in the incident power on the aircraft. In a future experiment, it would be helpful to have a direct measurement of the amplitude of the transmitted signal at the aircraft. This would at the very least indicate whether or not this is a significant factor, and if it is, it would help in working to incorporate the effect into the simulation.

A new set of measurements with improved signal-to-noise ratio would help significantly in determining whether the issue is the simulations or the measurements. Additional variations in the flight patterns and increasing the variation of the incidence and scattering angles would also help in the comparison, since larger variations in RCS should be more observable. It is also possible that improvements to the measurement processing algorithm could improve the RCS estimates. For example, an algorithm that reduces range walk (e.g., [41]) might be useful in reducing the spread of the received signal in the range-Doppler map and hence allow a more robust RCS estimate from the measurements.

Despite the limitations in the direct received-power comparisons, the simulated received-power levels have shown some skill in predicting whether or not a detection is possible. There are no detections for the low simulated received-power levels. It is clear that a high (simulated) bistatic RCS does not guarantee detection, but a low simulated RCS does guarantee a missed detection. This supports our contention that such an approach could usefully be applied to stealth aircraft to determine the likelihood of being able to detect them with a particular radar system. Doing so, however, would require good knowledge of both the geometry of the aircraft and the properties of any antireflection coating applied to such aircraft.

## Figures and Tables

**Table 1:** Main parameters of transmitter in Donaueschingen.

Parameter	Value
Location (WGS-84)	47°53′17″ N
	8°34′37″ E
DVB-T signal carrier frequency	482 MHz
Transmitting polarization	H
EIRP	46 dBW
Azimuth transmitting pattern $G_{tx}$	omnidirectional
Antenna height above ground	122 m
Ground elevation	917 m

**Table 2:** List of parameters of the analyzed DVB-T signal, as well as the parameters of the measurement campaign and signal processing.

Symbol	Description	Value
**DVB-T signals parameters**		
$f_{c}$	DVB-T signal carrier frequency	482 MHz
$T_{s}$	OFDM symbol duration	1120 µs
**Processing parameters**		
*B*	Signal bandwidth	9.142 MHz
$M_{s}$	Number of OFDM sysmbols per CPI	512
CPI	Coherent Processing Intervall	573 ms
$\Delta r_{B}$	Bistatic range resolution (c/B)	32.79 m
$\Delta f_{D}$	Bistatic Doppler resolution $\left(1/T_{s}\right)$	1.74 Hz

## Data Availability

Data sharing is not available for this article.
